# Potassium and Silicon Synergistically Increase Cadmium and Lead Tolerance and Phytostabilization by Quinoa through Modulation of Physiological and Biochemical Attributes

**DOI:** 10.3390/toxics10040169

**Published:** 2022-03-31

**Authors:** Hesham F. Alharby, Hassan S. Al-Zahrani, Ghulam Abbas

**Affiliations:** 1 Department of Biological Sciences, Faculty of Science, King Abdulaziz University, Jeddah 21589, Saudi Arabia; halharby@kau.edu.sa (H.F.A.); hsalzahrani@kau.edu.sa (H.S.A.-Z.); 2 Department of Environmental Sciences, COMSATS University Islamabad, Vehari Campus, Vehari 61100, Pakistan

**Keywords:** metal contamination, stomatal conductance, phytostabilization, oxidative stress, silicon

## Abstract

Cadmium (Cd) and lead (Pb) contaminated soils have increased recently, resulting in limited crop productivity. The ameliorative role of potassium (K) and silicon (Si) is well established in plants under heavy metals stress; however, their combined role under the co-contamination of Cd and Pb is not well understood. We hypothesized that the synergistic application of K and Si would be more effective than their sole treatment for increasing the Pb and Cd tolerance and phytostabilization potential of quinoa (*Chenopodium quinoa* Willd.). In the current study, quinoa genotype ‘Puno’ was exposed to different concentrations of Cd (0, 200 µM), Pb (0, 500 µM) and their combination with or without 10 mM K and 1.0 mM Si supplementation. The results revealed that the combined stress of Cd and Pb was more detrimental than their separate application to plant biomass (66% less than the control), chlorophyll content and stomatal conductance. Higher accumulation of Pb and Cd led to a limited uptake of K and Si in quinoa plants. The supplementation of metal-stressed plants with 10 mM K and 1.0 mM Si, particularly in combination, caused a significant increase in the growth, stomatal conductance and pigment content of plants. The combined stress of Cd and Pb resulted in an overproduction of H_2_O_2_ (11-fold) and TBARS (13-fold) and a decrease in membrane stability (59%). Oxidative stress induced by metals was lessened by 8-fold, 9-fold, 7-fold and 11-fold increases in SOD, CAT, APX and POD activities, respectively, under the combined application of K and Si. It is concluded that the exogenous supply of K and Si in combination is very promising for increasing Cd and Pb tolerance and the phytostabilization potential of quinoa.

## 1. Introduction

Soil contamination with heavy metals (HMs) has globally increased manyfold due to different anthropogenic activities, such as rapid urbanization, mining, agricultural practices and the release of effluents from industries [1,2]. For example, in China, the overall rate of HM contamination of soils has been reported to be 16.1% higher than the standards [3]. In America, the area of different sites polluted by HMs is almost 600,000 ha [4]. More than 200,000 sites have been reported as HM-contaminated sites in Sweden, France, Slovakia, Austria and Hungary, whereas approximately 10,000 sites have been reportedly contaminated with HMs in Poland and Greece [5].

Among various HMs, cadmium (Cd) is regarded as a non-essential element for plants, as it has no known function in plant physiology [6]. It is highly mobile in soil–plant systems, and is considered a highly toxic element for plants and humans [7,8]. Even if taken up in very small concentrations, Cd has negative effects on plant growth and physiology [6,9]. Lead (Pb) is another hazardous heavy metal having harmful effects on plants as well as humans [10,11]. Various anomalies, such as decline in pigments and photosynthetic attributes, limitation in essential nutrient uptake and decline in enzymatic activities, have been observed in plants exposed to Cd and Pb [6,10,12]. Furthermore, plants generate various reactive oxygen species when exposed to Cd and Pb stress [7,11,13]. Singlet oxygen (½O_2_), hydrogen peroxide (H_2_O_2_), hydroxyl free radicals (HO^•^) and superoxide (O_2_^•−^) are the major ROS that cause phytotoxicity in plants [11,14,15,16]. Cadmium- and Pb-induced ROS are detoxified within plant cells by various antioxidant enzymes and non-enzymes [10,11,17,18]. The antioxidant group of enzymes, including peroxidases (POD), superoxide dismutase (SOD) and catalases (CAT), has a crucial role in the detoxification of ROS within plants [6,10,12,18].

The remediation of metal-contaminated soils is crucial to sustaining a healthy environment. However, the removal of these heavy metals from the soil is very difficult due to their stability and nondegradable nature [1,2]. Among the different remediation techniques available, phytoremediation has been regarded as the most promising approach, due to its low cost, eco-friendliness, non-destructiveness and sustainability [5]. However, available data around the globe have revealed that the sole use of plants for soil remediation has limited success and often other amendments are needed to enhance the efficiency of the phytoremediation process [2]. 

The present climate change scenario warrants the use of plants that are tolerant to multiple environmental stresses for the sustainable utilization and rehabilitation of contaminated soils. Quinoa (*Chenopodium quinoa* Willd.) is the most exemplary plant in recent times, due to its potential role in food security and tolerance to multiple environmental stresses [12,13,14]. However, when quinoa is grown under high concentrations of various heavy metals, its growth and biomass are considerably decreased [12,13,17]. In order to increase the tolerance potential of plants to heavy metal toxicity, various amendments of organic and inorganic origin have been exogenously supplied to plants under stressful conditions [1,10,11,19,20].

Amongst macro nutrients, potassium (K) plays an indispensable part in the growth and normal metabolic activities of plants [21,22]. It takes part in many of the important physiological functions of plants, such as photosynthesis, enzyme activation, protein synthesis, cation–anion balance, stomatal regulation, osmoregulation and energy transmission [23,24]. Potassium supplementation increases plants’ growth and metal tolerance via limiting the accumulation of toxic metal ions and increasing enzyme activities in many plants [25,26].

Silicon (Si) is a beneficial element in plants, and is the second-most frequently occurring element in soil [19]. Its uptake by plants is crucial to increasing their growth and tolerance of many environmental stresses, i.e., drought, salinity and heavy metals [19,27,28]. Exogenous supply of Si mitigates heavy-metal-induced phytotoxicity in plants by improving plant–water relations and internal ionic balances, providing tolerance to oxidative stress and limiting the accumulation of toxic metal ions [29,30].

Therefore, the present study was carried out to unravel the effectiveness of K and Si on (a) the physiological and biochemical attributes of quinoa under Cd and Pb stress and (b) the Cd and Pb phytoremediation attributes of quinoa. The ameliorative role of both the K and Si is well established in plants under various heavy metal stresses; however, their combined role under the co-contamination of Cd and Pb is not well understood. This is the very first study that explores the possible mechanisms of concurrent application of K and Si in increasing the Cd and Pb tolerance potential of quinoa. Moreover, the study has crucial implications for the sustainable remediation of Cd and Pb contaminated soils using quinoa plants supplemented with K and Si.

## 2. Materials and Methods

### 2.1. Experimental Description

The current solution culture experiment was conducted on quinoa during 2020–2021. The average temperature of 10–26 °C and relative humidity around 55–72% was observed throughout the experiment. Quinoa genotype “Puno” was selected for this research because it is better adapted to the local climate and heavy metal stress [13]. Thirty days mature quinoa seedlings were taken from the nursery raised in sand culture and were grown in half-strength Hoagland’s nutrient solution [31] for one week before application of Pb and Cd treatments. Cadmium chloride and lead chloride salts were used in the nutrient medium for treatments of Cd (0, 200 µM), Pb (0, 500 µM) and their combination [12,13]. One week after the treatments’ application, plants under the combined application of Cd and Pb were supplied with 10 mM K (K_2_SO_4_) and 1.0 mM Si (Na_2_SiO_3_). The nutrient medium was supplied thorough aeration and weekly replacements. All the treatments were also applied in the fresh nutrient solution. The pH was stabilized around 6.5 ± 0.2 using either 0.1 N NaOH or HCl. There were four replications of each treatment with two plants per replicate.

### 2.2. Plant Sampling and Growth Measurements

Before harvesting, the plant samples were taken, immediately frozen and properly stored before biochemical analysis. The plants were harvested after four weeks of treatment exposure, and the roots were washed in 0.01 M HCl before being rinsed in distilled water for the removal of adsorbed Cd and Pb [16]. Root and shoot lengths were recorded with a meter scale. Air drying of the plant samples was done for 24 h before they were oven-dried at 70 °C for 48 h to record their dry weights.

### 2.3. Determination of Metal Contents 

Roots and shoots of quinoa were crushed separately using a porcelain grinder, and were digested in HClO_4_ and HNO_3_ taken in 1:2 ratio, respectively. After digestion, samples were filtered and the concentration of K and Na was measured using a flame photometer (BWB-XP5). The concentration of Si in plant samples was analyzed as detailed by [30] through UV–vis spectrophotometer (Lambda 25, PerkinElmer, Inc., Los Angeles, CA, USA) at 700 nm wavelength. The concentrations of Cd and Pb were determined using an atomic absorption spectrophotometer (PinAAcle 900F, PerkinElmer, Inc. Los Angeles, CA, USA. PerkinElmer single-element calibration standards were used as the stock standards. Working standards were prepared by serial dilution of stock standards. Type I Millipore water acidified with 0.2% Suprapur nitric acid (Merck^®^, Darmstadt, Germany) was used for the calibration of blanks and for all the dilutions. Certified reference material GBW 10016 in tea leaves was used for the quality control in the determination of both metals. The detection limits for Cd and Pb were 0.002 μg L^−1^ and 0.04 μg L^−1^, respectively. Recoveries of Cd and Pb were 98% and 99%, respectively. The standard deviations for Cd and Pb were maintained at 0.002 and 0.2, respectively. A graphite furnace accessory with the following conditions was used for the determination of Cd and Pb, respectively: wavelengths 228.8 and 283.3 nm; slit 0.7 and 0.7 nm; standards 0.2, 0.5, 1.0 and 5.0, 10, 25 μg L^−1^. The matrix modifier (5 μL) for both Cd and Pb was 0.05 mg NH_4_H_2_PO_4_ and 0.003 mg Mg(NO_3_)_2_; the read time for both Cd and Pb was 5 s; the correlation coefficients for Cd and Pb were 0.9997 and 0.9991; and sample volume for both Cd and Pb was 20 μL.

### 2.4. Leaf Pigments and Stomatal Conductance

The procedure described by Lichtenthaler (1987) [32] was used to evaluate all kinds of chlorophylls, including Chl a, Chl b and total Chl. Leaves (0.1 g) were immersed in liquid nitrogen for a while to stop their metabolic activity and crushed in 80% hydro-acetone. Afterward, the samples were centrifuged at 3000 rpm (10 min). A UV–vis spectrophotometer (Lambda 25, PerkinElmer, Inc., Los Angeles, CA, USA) was used to measure the absorbance of the collected supernatant at 663.2 and 646.8 nm wavelengths. The stomatal conductance of the second top leaf of each plant was estimated at 10 a.m. to 2 p.m. on a sunny day with the help of a portable leaf porometer (Decagon Devices, Pullman, WA, USA).

### 2.5. Oxidative Stress Attributes

The method of [33] was used to determine hydrogen peroxide (H_2_O_2_) contents. For this purpose, 0.1% trichloroacetic acid was used for the homogenization of 0.5 g of leaf samples. Then, samples were centrifuged at 12,000× *g* for 20 min. The reaction mixture (pH 7.0) consisted of 1 mL of potassium phosphate buffer (10 mM), potassium iodide (1 mL, 2 M) and leaf extract (1 mL). The concentration of H_2_O_2_ in the sample was estimated on a UV–vis spectrophotometer at a wavelength of 390 nm. The assay proposed by [34] was used for the determination of lipid peroxidation (thiobarbituric acid reactive substances, TBARS). The membrane stability index (MSI) of leaf samples was determined as proposed by [35].

### 2.6. Enzymatic Activities

Antioxidant enzymes were measured in the most recently appearing leaves of quinoa plants under liquid nitrogen. Leaf samples (0.5 g) were ground in 0.1 M phosphate buffer solution with pH 7. The leaf extract of ground samples was obtained after centrifugation at 15,000× *g* for 30 min at 4 °C temperature. The methodology proposed by [36] was used for calculating the SOD enzymatic activity, which involved a 50% decrease in nitroblue tetrazolium (NBT). The APX activity was measured using the procedure proposed by [37], and was presented as µM of ascorbate min^−1^ mg^−1^ protein. CAT activity was determined as described by [38] and expressed as µM of H_2_O_2_ tainted min^−1^ mg^−1^ protein. The activity of peroxidase (POD) was estimated using the method proposed by [39] and given as µM guaiacol oxidized min^−1^ mg^−1^ protein.

### 2.7. Metal Accumulation and Translocation

The bioconcentration factor (BCF) and translocation factor (TF) were calculated as proposed by [12]. The BCF was measured in terms of ratio of concentrations of metals (Cd and Pb) in plants vs. solution, whereas the concentration ratio of metals between roots and shoots was measured for the determination of the TF. Dry weights of metal-stressed plants were divided by the dry weights of control plants for tolerance index (TI).

### 2.8. Statistical Analyses

A completely randomized design (CRD) was adopted for the present experiment. The data were analyzed statistically by a one-way ANOVA (analysis of variance) using Statistix 8.1 statistical software. Treatments were compared at a 5% probability level by least significant difference test [40].

## 3. Results

### 3.1. Plant Growth

The growth of quinoa plants was decreased more under Cd than Pb stress; however, the highest decrease was found under the combined application of 200 µM Cd and 500 µM Pb, as shown in Table 1. It was noticed that when Cd and Pb were applied together, the dry weights of shoot and root were respectively 66% and 67% lower than that of the control. Under the combined treatment of Cd and Pb, shoot and root lengths were 63% and 68% lower than those of the control plants. The supplementation of metal-stressed plants with 10 mM K was more effective than 1.0 mM Si in reducing metal toxicity. However, the combined application of both the Si (1.0 mM) and K (10 mM) was found to be the most promising treatment in the amelioration of Cd and Pb stress in quinoa plants. Shoot and root dry weights and shoot and root lengths were only 16%, 17%, 16% and 15% lower than those of the control under the combined application of Si and K to the metal-stressed plants.

### 3.2. Pigment Contents and Stomatal Conductance 

Pigments and stomatal conductance were decreased more when plants were exposed to Cd as compared to Pb in the growth medium (Table 2). Chl a, Chl b, total Chl contents and stomatal conductance were 62%, 66%, 64% and 70% less than those of the control under 200 µM Cd + 500 µM Pb treatment. These attributes were improved more under 10 mM K compared to 1.0 mM Si supplied to metal-stressed plants. The declines in Chl a, Chl b, total Chl contents and stomatal conductance were 14%, 13%, 14% and 15%, respectively, when plants were grown under a 200 µM Cd + 500 µM Pb treatment with the addition of Si and K together.

### 3.3. Oxidative Stress Attributes

**Figure 1:**
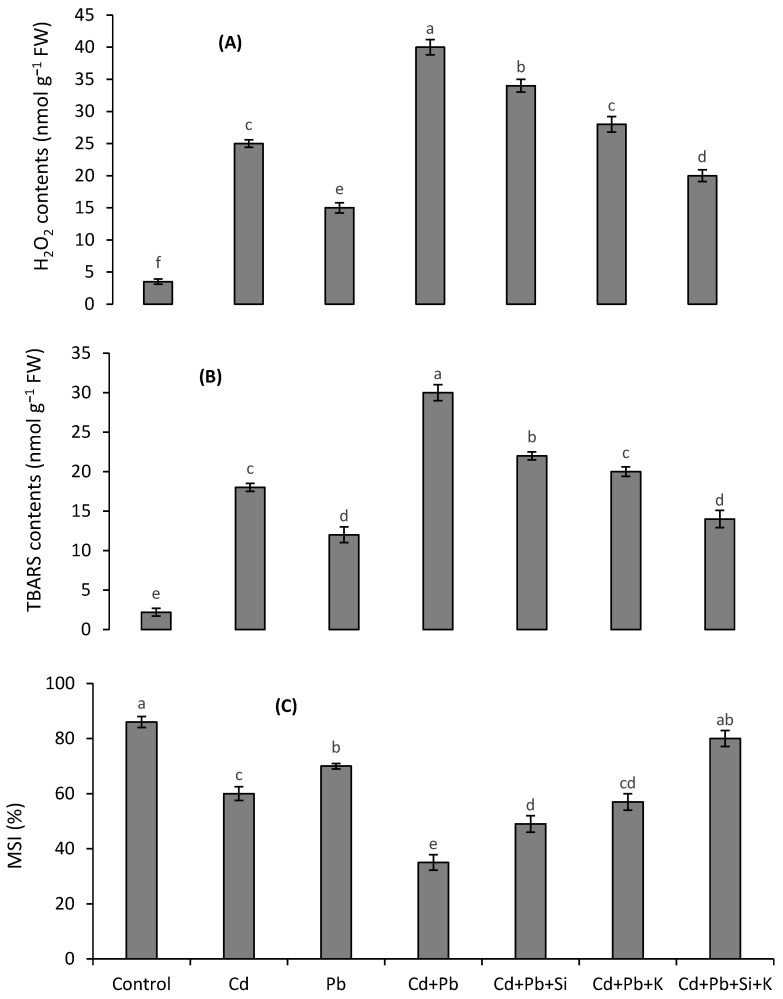
Effects of different Cd and Pb treatments on H_2_O_2_ contents (**A**), TBARS contents (**B**) and MSI (**C**) on quinoa with and without supplementation with Si and K. Bars with different letters indicate a significant difference among the treatments at 5% significance level.

The generation of H_2_O_2_ and resultant oxidative stress (lipid peroxidation) were increased more under Cd than Pb stress and to a greater extent under their combined treatment (Figure 1A,B). Oxidative stress was decreased more under 10 mM K than 1.0 mM Si application. When Si and K were applied together, H_2_O_2_ and TBARS contents were 2-fold and 2.1-fold lower than the combined stress of both metals. The MSI was decreased by 59% under Pb and Cd combination (Figure 1C). The MSI was decreased by only 7% in the combined stress of Cd and Pb with Si and K amendments.

### 3.4. Antioxidant Enzymes

**Figure 2:**
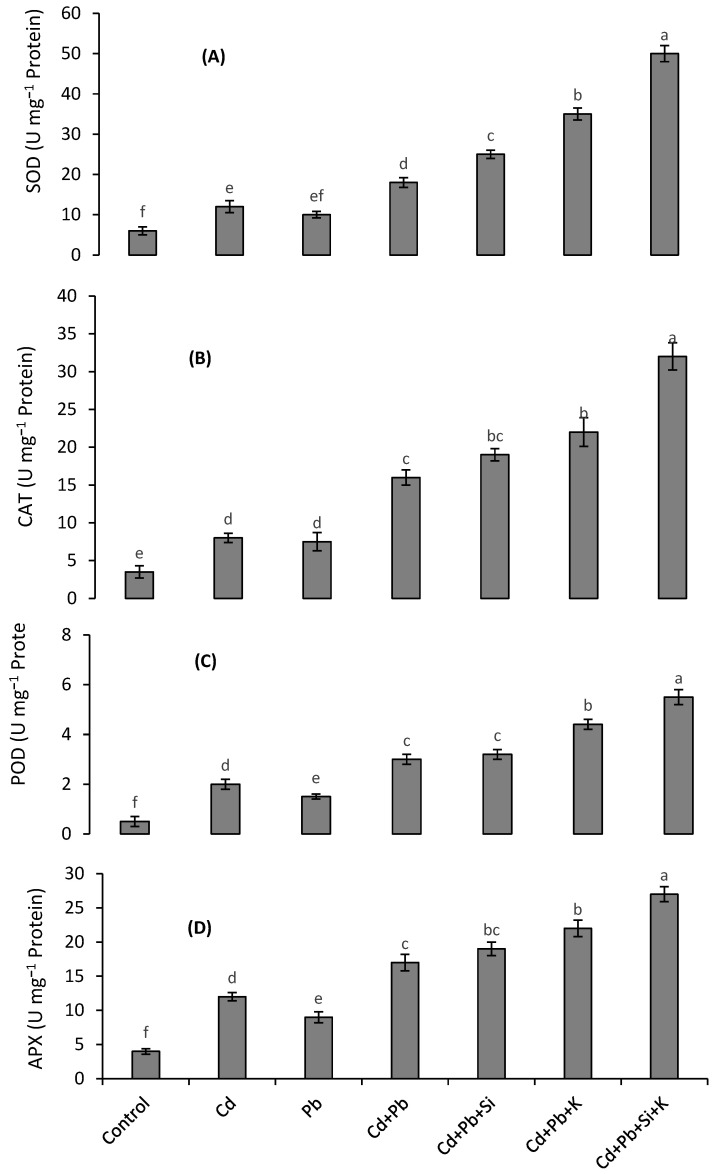
Effects of different Cd and Pb treatments on the activities of SOD (**A**), CAT (**B**) POD (**C**) and APX (**D**) on quinoa with and without supplementation with Si and K. Bars with different letters indicate the significant difference among the treatments at 5% significance level.

The activities of antioxidant enzymes showed an increasing trend under Pb and Cd toxicity; however, the combined application of both metals resulted in the highest increase in the activities of enzymes (Figure 2A–D). The activities of SOD, CAT, APX and POD were 3-fold, 4.5-fold, 4-fold and 6-fold, respectively, higher than those in the control under the combined stress of both metals. The addition of K was more effective than Si in increasing the activities of antioxidants. Under the joint treatment of both metals, the exogenous supply of 1.0 mM Si and 10 mM K resulted in 8-fold, 9-fold, 7-fold and 11-fold higher activities of SOD, CAT, APX and POD, respectively.

### 3.5. Silicon and K Concentrations

**Figure 3:**
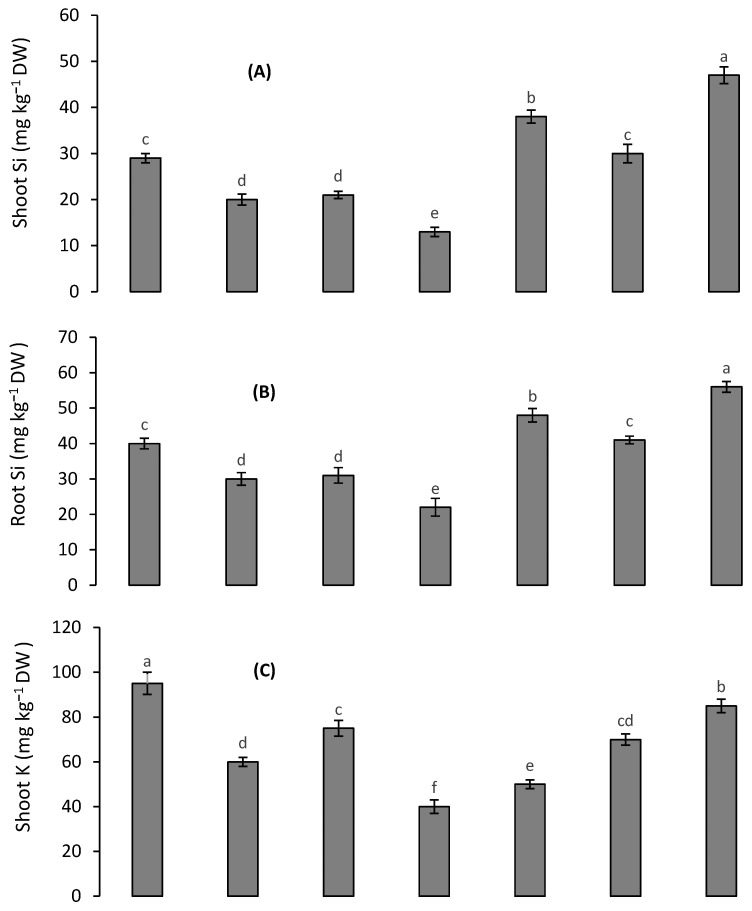

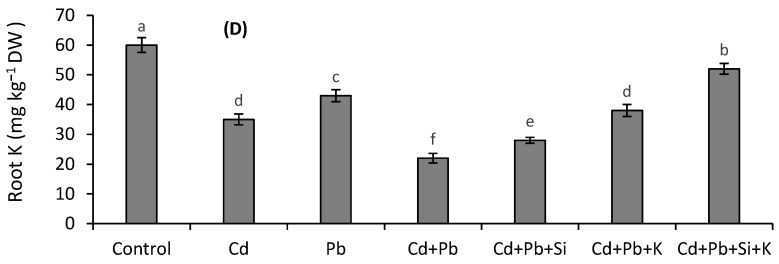
Effect of different Cd and Pb treatments on shoot Si (**A**), root Si (**B**) shoot K (**C**) and root K (**D**) concentrations in quinoa with and without supplementation with Si and K. Bars with different letters indicate the significant difference among the treatments at 5% significance level.

Root and shoot Si accumulation was decreased to a similar extent under Cd and Pb stress; however, their combined application caused the lowest accumulation of Si in quinoa plants (Figure 3A,B). The accumulation of K was decreased more in the root and shoot of quinoa plants in the presence of Cd than Pb in the medium; however, the combined supplication of both metals caused the highest reduction in K accumulation in the plants’ tissues (Figure 3C,D). The accumulation of K and Si was increased under the respective application of 10 mM K and 1.0 mM Si. The highest accumulations of both elements were observed under the combined treatment with 1.0 mM Si and 10 mM K.

### 3.6. Metal Accumulation and Translocation

**Figure 4:**
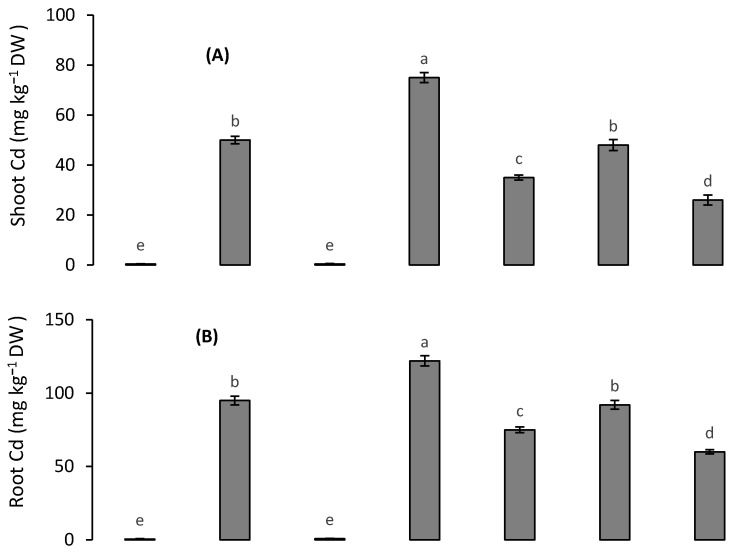

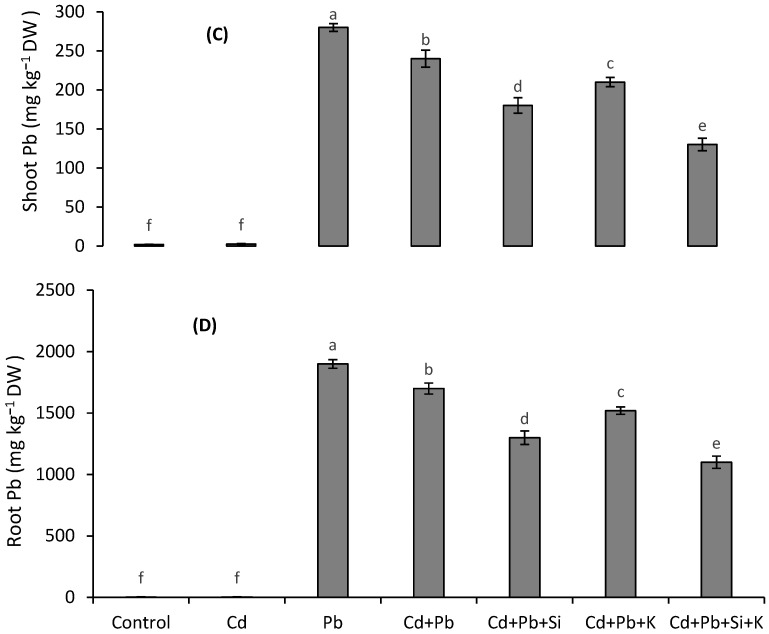
Effect of different Cd and Pb treatments on shoot Cd (**A**), root Cd (**B**) shoot Pb (**C**) and root Pb (**D**) concentrations in quinoa with and without supplementation with Si and K. Bars with different letters indicate the significant difference among the treatments at 5% significance level.

The accumulation of Cd and Pb in the root and shoot of quinoa was increased under Cd and Pb treatments. When 200 µM Cd and 500 µM Pb were applied together, Cd accumulation in root and shoot was even more increased but Pb accumulation was decreased (Figure 4A–D). The addition of 1.0 mM Si resulted in a greater decrease in Cd and Pb accumulation in root and shoot than with the addition of 10 mM K. However, the combined treatment with both Si and K caused the lowest uptake of Cd and Pb by quinoa tissues. The values of the BCF were greater than one for both Cd and Pb (Table 3). However, the values of the TF were less than one for both Cd and Pb. The value of the TI for Pb alone was greater than that with the Cd alone treatment. However, the lowest value of the TI was observed under the 200 µM Cd + 500 µM treatment (Table 3). The addition of 10 mM K resulted in a greater increase in the TI than did 1.0 mM Si. However, the highest value of the TI was observed under the combined treatment with Si and K.

### 3.7. Multivariate Comparison of Variables

**Figure 5:**
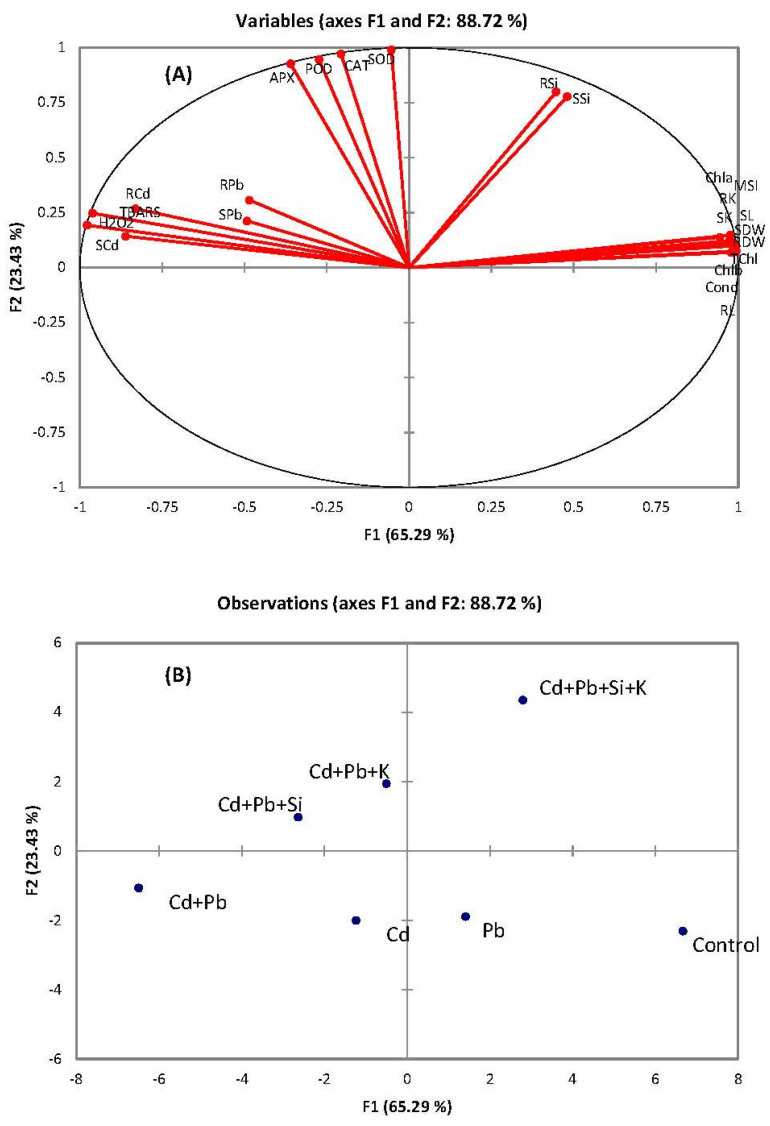
Principal component analysis of plant growth attributes (**A**) and treatments (**B**) of quinoa exposed to Cd and Pb treatments with and without supplementation of Si and K.

The associations among different variables were determined by principal component analysis (PCA) and Pearson correlations (Figure 5A,B; Table 4). The PCA grouped the response variables into six factors, viz., F1 to F6. However, only four factors contributed mainly. These four factors exhibited 65%, 23%, 8% and 2%, variability, respectively. Close associations were noticed among four groups of variables: (a) growth and physiological attributes, (b) Si contents in root and shoot, (c) antioxidant enzymes and (d) Pb and Cd contents and oxidative stress. The PCA also divided different treatments into various axes. Cadmium and Pb treatments were scattered along different axes when supplemented with or without Si and K. The control treatment alone was on the right of the F1 axis and was characterized by higher growth attributes, pigments and stomatal conductance. The combined Pb + Cd treatment was at the opposite end of this axis and characterized by high oxidative stress and metal contents. The addition of K or Si groups together was more related to high antioxidant enzymes, while Si + K was at the top of the F2 axis and related with the highest Si and K contents, highest enzyme activities and lowest metal contents.

Pearson correlations indicated strong positive correlations of plant growth attributes with pigments, stomatal conductance, and root and shoot K contents, and strong negative correlations with shoot Cd and oxidative stress attributes. Pigments and stomatal conductance had strong positive correlations with root and shoot K contents and strong negative correlations with oxidative stress attributes and root and shoot Cd contents. Root and shoot Cd contents had strong positive correlations with oxidative stress attributes and strong negative correlations with root and shoot K contents (Table 4).

## 4. Discussion

Contamination of soil with heavy metals, particularly Cd and Pb, has increased worldwide tremendously during the last two decades [2]. The threshold levels for these metals have been crossed in many countries of the world [4,5]. This alarming increase in soil contamination globally has been attributed to different anthropogenic activities, such as mining, rapid urbanization, faulty agricultural practices and the release of effluents from industries [1,10].

For sustaining a healthy environment, the remediation of heavy-metal-contaminated sites is indispensable. However, these heavy metals are very durable, stable and nondegradable; hence, their removal from the soil is very difficult [1,2]. Among the different remediation techniques employed, phytoremediation has been regarded as the most promising approach due to its low cost, eco-friendliness, non-destructiveness, and sustainability [5]. However, available data around the globe have revealed that the sole use of plants for soil remediation has had limited success, and that often other amendments are needed to enhance the phytoremediation efficiency of the plants [2].

The present research work was undertaken to explore the role of the exogenous supply of Si and K on the Cd and Pb tolerance and phytoremediation potential of quinoa plants. The growth and biomass of quinoa plants greatly reduced when Cd and Pb were applied in combination. In line with our findings, reductions in the growth of many plants, including quinoa, have been reported by different researchers for Cd [6,9,12] and Pb [10,11,16]. Cadmium- and Pb- induced reduction in plant growth may be due to restrictions on essential nutrient uptake, chlorophyll biosynthesis, stomatal conductance and the generation of various ROS [6,7,10,41,42]. When Si and K were applied to Cd-and Pb-stressed plants, plant growth of quinoa was significantly improved. It was particularly observed under the combined treatment with Si and K. Silicon has many positive effects on plants under metal stress conditions [29,43]. In line with our findings, Huang et al. [44] reported that the shoot and root growth of Cd-stressed rice was increased under Si application. In another study on wheat, it was reported that plant growth of Cd- and Pb- stressed wheat was considerably increased with the exogenous application of Si [30]. Growth enhancement under Si application may be due to the increased uptake and utilization of plant nutrients [45]. Findings have indicated that Si can enhance the resistance of plants to environmental stresses due to specific cell formation in cell walls leading to increased nutrient absorption [46]. In addition, Si can precipitate heavy metals; hence, their availability and plant uptake are reduced, which results in the enhancement of plant growth attributes [44,47].

On the other hand, K has an indispensable role in plants under stressful conditions [23]. In the current study, it was observed that K treatment either alone or in combination with Si increased the plant growth of quinoa suffering from Cd and Pb stress. The enhanced plant growth in the presence of K has been attributed to an increased nutrient and water uptake by quinoa plants as well as increased photosynthetic activity and other metabolic processes [24,26]. The combined application with both Si and K was the most potent treatment in the alleviation of Cd and Pb toxicity in quinoa plants. Similar to our results, it was observed that combined treatment with Si and K was more efficient than their sole application for the alleviation of Cd stress in *Gladiolus grandiflora* L. [26].

Both the Cd and Pb treatments caused considerable reductions in pigment contents and the stomatal conductance of quinoa plants. The combined application of both metals was even more detrimental to these attributes. Previously, many studies found that both Cd and Pb caused phytotoxicity to pigments and the stomatal conductance of various plants [6,7,16]. The reasons for the reduction in pigments under Cd and Pb exposure include the destruction of the chlorophyll structure and direct toxicity of ROS [11,48,49]. Non-stomatal limitations of photosynthesis induced by Cd and Pb caused the decline in stomatal conductance in quinoa leaves [13,50]. The application of Si to quinoa plants caused significant increases in their chlorophyll contents and stomatal conductance. In accordance with our results, it has been reported that Si supply can efficiently lessen Cd and Pb toxicity and increase chlorophyll contents and gaseous exchange [30,50]. The addition of K alone as well as in combination with Si causes a remarkable increase in chlorophyll contents and stomatal conductance, as has been noticed by [26]. According to Song et al. [51], K supplementation in plants facing heavy metal stress restricts the biodegradation of photosynthetic pigments and improves the photosynthetic process. The higher increase in these attributes under the combined application of Si and K may be due to better protection from ROS and a limited accumulation of toxic metal ions [26,27].

Quinoa plants experienced severe oxidative stress due to the generation of H_2_O_2_. Due to the overproduction of H_2_O_2_, TBARS contents were increased, causing a decrease in cell membranes’ stability. The generation of H_2_O_2_ and subsequent damage to cell membranes were more harmful under the concurrent application of both metals. Cd- and Pb-induced oxidative stress and membrane damage has been noticed in many plants [6,10,16]. H_2_O_2_ has the tendency to convert into other more toxic ROS, such as hydroxyl radicals; therefore, it is considered the most crucial ROS [7,11]. Hence, for the protection of metal-stressed plants from the toxicity of ROS the mitigation of H_2_O_2_ is essential. The detoxification of ROS within plant cells is carried out by various antioxidant enzymes and non-enzymes [6,18]. SOD is known as the most important enzyme, as it converts superoxide radicals into H_2_O_2_ and oxygen [7,16,23]. We observed a considerable increase in SOD activity in quinoa upon the dual stress of Cd and Pb. The exogenous supply of Si and K together further increased the SOD activity in current study. Si- and K-induced increase in the activity of SOD in plants facing Cd and Pb toxicity has been noticed previously [27,30]. The enhanced activity of SOD may be due to the direct interaction of both metals with it or because of its involvement in the conversion of superoxide into H_2_O_2_ [29,52]. The H_2_O_2_ is further detoxified into oxygen and water by peroxidases (POD) and catalases (CAT) [11,18,30]. The outcomes of the current study indicate that the activities of CAT, APX and POD showed an increasing trend on Cd and Pb toxicity. The addition of Si and K jointly further enhanced the activity of these enzymes, as has been observed in previous research [23,27]. Hence, it can be concluded that both Si and K synergistically played their role in alleviation of oxidative stress in quinoa though the overactivation of antioxidant enzyme system.

The K concentration was decreased in the root and shoot of quinoa in the presence of Cd and Pb, which is in line with previous studies [16,18]. Potassium plays many crucial functions in plant physiology, i.e., enzyme activation, chlorophyll synthesis, osmotic adjustment, cell enlargement, the maintenance of membrane potential and cytoplasmic pH [23,53]. Therefore, maintenance of higher cellular K levels is directly related to higher metal tolerance in plants [12,16]. We noticed that higher accumulation of Cd and Pb resulted in the reduced absorption of K by root of quinoa [18,29]. The competition of Cd and Pb with K for the same cations channels resulted in the less uptake of K by quinoa [18,48,54]. Additionally, it has been observed that both Cd and Pb interfere with the absorption and translocation of many nutrients, including K, resulting in their deficiency in plants [16,55]. Supplementation of Cd- and Pb-stressed plants with K resulted in an increased accumulation of K in quinoa and caused a considerable decrease in the accumulation of heavy metal ions [26]. This decrease in Cd and Pb uptake in the presence of K is due to the substitution of metal ions with K [55], exclusion of Cd and Pb ions [56,57] and retention of Cd and Pb ions in proteins and other channel blockers [26,57]. Similar to K, the concentration of Si was also decreased in plant tissues under Cd and Pb stress, and the exogenous supply of Si caused a significant increase in the Si content in plants tissues [30,58,59]. The accumulation of both Cd and Pb was decreased with an increase in Si uptake. This limited uptake of both Cd and Pb might be due to the excessive deposition and translocation of Si within root to shoot [30]. The augmented deposition of Si in roots and its increased uptake is regarded as an important mechanism for restricting the channels of Cd and Pb ions uptake. Additionally, Si forms stable complexes and precipitates of metals ions as a cofactor that are not taken up by plants [60,61]. Consequently, the enhanced accumulation of Si in quinoa tissues can be linked with the decrease in Cd and Pb accumulation and toxicity. In line with our results, it was found that the accumulation of Si in the endodermis of roots is the main mechanism that physically obstructed the apoplastic bypass flow within roots, leading to a limited transport of metal ions [27,62]. The abovementioned mechanisms were even more noticeable under the combined treatment of both Si and K; hence, the accumulation of both Cd and Pb was severely decreased. We noticed that in the combined treatment of Cd and Pb the accumulation of Cd by quinoa was increased, whereas the accumulation of Pb was decreased. This indicated the greater availability and mobility of Cd than Pb under the prevalent range of the pH of the medium [16,63].

In the present study, the roots of quinoa retained more Cd and Pb and less was transferred to shoots. It has been reported that various phytochelatins (PCs) are produced in roots and metal ions form complexes with them. These PCs are mainly sequestered within vacuoles of root, leading to less transport of Cd and Pb from root to shoot [6,11,12,49].

In the present study, the BCF for both Cd and Pb were greater than one, whereas, the TF values were less than one for both metals. Root-to-shoot translocation for both metals was less than one for both the single and combined application of Pb and Cd. Similar to these results, less transfer of Pb and Cd from the root to the aerial parts has been noticed in plants in both soil and hydroponic conditions [11,12,16]. The supplementation of metal-stressed plants with Si and K alone as well as in combination further decreased the BCF and TF of both metals. The collective application of Pb and Cd indicated the lowest value of TI, which was increased with the addition of K and Si. However, the combined treatment with K and Si caused the highest value of the TI in quinoa. On the basis of the BCF, TF and TI, it is concluded that quinoa is a promising plant for the phytostabilization of both Cd and Pb in contaminated soils. Moreover, the addition of Si and K in combination is a very suitable approach for increasing Cd and Pb tolerance and restricting their uptake and root-to-shoot translocation in quinoa.

Different response variables and treatments were compared by PCA. As mentioned in previous studies, this data analysis technique is considered the most appropriate to understanding the relationships among different variables [12]. The PCA indicated that the oxidative stress indictators (H_2_O_2_, MDA, MSI) and metal contents in the shoot and root were clustered closely. Similarly, the growth and physiological attributes of quinoa were grouped closely. This close association was also confirmed through Pearson correlation, which indicated strong negative correlations of growth and physiological attributes with Pb and Cd accumulation by quinoa. Similarly, oxidative stress attributes (H_2_O_2_, TBARS, MSI) had strong negative correlations with plant growth and physiological attributes. Shoot and root K contents had stronger positive correlations than Si contents with the growth attributes of quinoa. The clustering of different treatments shows that the combination of Cd and Pb was the most detrimental treatment, compared to their separate application, to plant growth and physiology. Hence, it stayed negative in both x- and y-axes. The placement of the combined treatment of Si and K in the positive x- and y-axes indicates that the combined application of both Si and K was more beneficial than their sole applications for quinoa plants suffering from Cd and Pb stress.

## 5. Conclusions

The contamination of soils with Cd and Pb is a global issue, and has increased to alarming levels due to various anthropogenic activities. Among the different available options for the remediation of metal contaminated soils, phytoremediation is considered as the most promising and sustainable approach. Different plant species have been used for the phytoremediation of contaminated soils, but the use of plants without any organic or inorganic amendments for soil remediation has had limited success. The results of the current study indicate that the combined application of 10 mM K and 1.0 mM Si was the most promising treatment for enhancing the Cd and Pb tolerance of quinoa. Limited translocation of Cd and Pb to the above-ground parts under the exogenous supply of K and Si suggest that quinoa is suitable for the phytostabilization of Cd and Pb contaminated soils. Nevertheless, further research is warranted to determine the effectiveness of K and Si on the metal uptake and portioning in quinoa and other plants growing on Cd and Pb co-contaminated soils.

## Figures and Tables

**Table 1:** Growth attributes of quinoa plants under Cd and Pb treatments with or without supplementation with Si and K.

Treatment	Shoot Length (cm)	Root Length (cm)	Shoot Dry Weight (g plant^−1^)	Root Dry Weight (g plant^−1^)
Control	20.3 ± 0.5 a	19 ± 0.6 a	1.8 ± 0.05 a	0.40 ± 0.01 a
Cd	12.1 ± 0.2 d	12 ± 0.2 d	1.05 ± 0.02 d	0.24 ± 0.007 d
Pb	14.2 ± 0.5 c	13 ± 0.5 cd	1.3 ± 0.03 c	0.27 ± 0.013 cd
Cd + Pb	7.5 ± 0.2 e	6.0 ± 0.2 f	0.6 ± 0.04 e	0.13 ± 0.01 f
Cd + Pb + Si	12.2 ± 0.2 d	10 ± 0.5 e	1.0 ± 0.03 d	0.21 ± 0.008 e
Cd + Pb + K	15.0 ± 0.4 c	14 ± 0.4 c	1.25 ± 0.04 c	0.29 ± 0.011 c
Cd + Pb + Si + K	17.1 ± 0.6 b	16 ± 0.6 b	1.5 ± 0.05 b	0.33 ± 0.01 b

Data are the average values of four replicates followed by standard error. Different letters in each column represent the significant difference at 5% significance level.

**Table 2:** Pigment contents and stomatal conductance of quinoa plants under Cd and Pb treatments with and without supplementation of Si and K.

Treatment	Chl a (µg g^−1^ FW)	Chl b (µg g^−1^ FW)	Total Chl (µg g^−1^ FW)	Stomatal Conductance (mmol m^−2^ s^−1^)
Control	180 ± 5 a	250 ± 5 a	430 ± 10 a	480 ± 10 a
Cd	105 ± 4 d	125 ± 8 d	230 ± 12 d	270 ± 12 d
Pb	130 ± 5 c	190 ± 8 c	320 ± 8 c	350 ± 8 c
Cd + Pb	60 ± 4 e	94 ± 8 e	154 ± 15 e	145 ± 15 f
Cd + Pb + Si	92 ± 6 de	110 ± 6 d	202 ± 12 de	230 ± 12 e
Cd + Pb + K	120 ± 5 c	188 ± 5 c	305 ± 10 c	300 ± 10 cd
Cd + Pb + Si + K	155 ± 4 b	215 ± 4 b	370 ± 12 b	410 ± 12 b

Data are the average values of four replicates followed by standard error. Different letters in each column represent the significant difference at 5% significance level.

**Table 3:** Bioconcentration factor (BCF), translocation factor (TF) and tolerance index (TI) of quinoa plants under Cd and Pb treatments with and without supplementation with Si and K.

Treatment	Pb		Cd		
	BCF	TF	BCF	TF	TI
Cd	-	-	2.23 ± 0.25 b	0.53 ± 0.05 b	58 ± 2.1 c
Pb	2.70 ± 0.3 a	0.15 ± 0.005 a	-	-	72 ± 3.0 b
Cd + Pb	2.31 ± 0.2 b	0.14 ± 0.006 b	3.35 ± 0.2 a	0.61 ± 0.02 a	33 ± 2.5 d
Cd + Pb + Si	1.73 ± 0.1 d	0.13 ± 0.005 b	1.56 ± 0.2 c	0.46 ± 0.03 d	55 ± 1.9 c
Cd + Pb + K	2.02 ± 0.2 b	0.13 ± 0.003 b	2.14 ± 0.15 b	0.52 ± 0.02 bc	69 ± 2.0 b
Cd + Pb + Si + K	1.25 ± 0.1 e	0.11 ± 0.003 c	1.16 ± 0.1 d	0.43 ± 0.01 e	83 ± 2.1 a

Data are the average values of four replicates followed by standard error. Different letters in each column represent the significant difference at 5% significance level.

**Table 4:** Pearson correlation matrix of different attributes of quinoa under Cd and Pb in the presence of Si and K. Values in bold indicate a significant relationship among different variables.

Variables	RDW	SDW	Chla	Chlb	TChl	Cond	SOD	CAT	POD	APX	H_2_O_2_	TBARS	MSI	RCd	SCd	SK	RK	SPb	RPb	RSi
SDW	**0.9925**																			
Chla	**0.9553**	**0.9585**																		
Chlb	**0.9873**	**0.9950**	**0.9652**																	
TChl	**0.9761**	**0.9820**	**0.9935**	**0.9887**																
Cond	**0.9774**	**0.9904**	**0.9614**	**0.9983**	**0.9862**															
SOD	0.0670	0.0421	0.1005	0.0547	0.0786	0.0270														
CAT	−0.0963	−0.1134	−0.0495	−0.1002	−0.0741	−0.1250	**0.9824**													
POD	−0.1478	−0.1791	−0.1025	−0.1655	−0.1338	−0.1930	**0.9730**	**0.9841**												
APX	−0.2400	−0.2675	−0.2084	−0.2582	−0.2348	−0.2851	**0.9501**	**0.9793**	**0.9923**											
H_2_O_2_	**−0.9019**	**−0.9244**	**−0.8888**	**−0.9322**	**−0.9168**	**−0.9451**	0.2903	0.4321	0.4873	0.5716										
TBARS	**−0.9260**	**−0.9490**	**−0.8821**	**−0.9476**	**−0.9191**	**−0.9565**	0.2495	0.3926	0.4578	0.5345	**0.9900**									
MSI	**0.9471**	**0.9644**	**0.9205**	**0.9808**	**0.9555**	**0.9875**	0.0282	−0.1242	−0.1874	−0.2768	**−0.9402**	**−0.9490**								
RCd	−0.7228	**−0.7926**	**−0.7572**	**−0.7854**	**−0.7790**	**−0.8099**	0.3429	0.4350	0.5242	0.5800	**0.8926**	**0.8899**	**−0.8010**							
SCd	**−0.7673**	**−0.8348**	**−0.7714**	**−0.8224**	**−0.8023**	**−0.8422**	0.2256	0.3295	0.4203	0.4744	**0.8843**	**0.9006**	**−0.8409**	**0.9839**						
SK	**0.9768**	**0.9820**	**0.9831**	**0.9925**	**0.9957**	**0.9919**	0.0683	−0.0899	−0.1429	−0.2444	**−0.9296**	**−0.9302**	**0.9746**	**−0.7770**	**−0.8051**					
RK	**0.9704**	**0.9779**	**0.9640**	**0.9932**	**0.9858**	**0.9950**	0.0326	−0.1176	−0.1835	−0.2797	**−0.9385**	**−0.9419**	**0.9820**	**−0.7758**	**−0.7991**	**0.9912**				
SPb	−0.5084	−0.4574	−0.2852	−0.4647	−0.3642	−0.4540	0.2144	0.3111	0.3177	0.3377	0.4667	0.5092	−0.4830	0.1254	0.1637	−0.4038	−0.4751			
RPb	−0.4906	−0.4420	−0.2677	−0.4465	−0.3463	−0.4382	0.3096	0.4045	0.4063	0.4239	0.4832	0.5209	−0.4664	0.1543	0.1815	−0.3867	−0.4568	**0.9947**		
RSi	0.5461	0.5556	0.4590	0.5339	0.4928	0.5101	0.7044	0.6313	0.5450	0.5230	−0.2378	−0.3385	0.5010	−0.2257	−0.3733	0.4831	0.4759	−0.1747	−0.0983	
SSi	0.5085	0.5206	0.4278	0.5038	0.4622	0.4819	0.7301	0.6647	0.5760	0.5564	−0.2044	−0.3030	0.4789	−0.1985	−0.3460	0.4539	0.4504	−0.1543	−0.0748	**0.9975**

The values in bold represent the significant correlations among the variables.

## Data Availability

Data will be available as requested.

## References

[1] Palansooriya K.N., Shaheen S.M., Chen S.S., Tsang D.C.W., Hashimoto Y., Hou D., Bolan N.S., Rinklebe J., Ok Y.S. (2020). Soil amendments for immobilization of potentially toxic elements in contaminated soils: A critical review. Environ. Int..

[2] Liu K., Guan X., Li C., Zhao K., Yang X., Fu R., Li Y., Yu F. (2022). Global perspectives and future research directions for the phytoremediation of heavy metal-contaminated soil: A knowledge mapping analysis from 2001 to 2020. Front. Environ. Sci. Eng..

[3] Ministry of Environmental Protection and Ministry of Land and Resources of China (2014). Bulletin of National Soil Pollution Survey. http://www.zhb.gov.cn/gkml/hbb/qt/201404/W020140417558995804588.pdf.

[4] Mahar A., Wang P., Ali A., Awasthi M.K., Lahori A.H., Wang Q., Li R., Zhang Z. (2016). Challenges and opportunities in the phytoremediation of heavy metals contaminated soils: A review. Ecotoxicol. Environ. Saf..

[5] Yadav K.K., Gupta N., Kumar A., Reece L.M., Singh N., Rezania S., Khan S.A. (2018). Mechanistic understanding and holistic approach of phytoremediation: A review on application and future prospects. Ecol. Engin..

[6] Rasafi T.E., Oukarroum A., Haddioui A., Song H., Kwon E.E., Bolan N., Tack F.M., Sebastian A., Prasad M.N.V., Rinklebe J. (2021). Cadmium stress in plants: A critical review of the effects, mechanisms, and tolerance strategies. Crit. Rev. Environ. Sci. Technol..

[7] Zhao H., Guan J., Liang Q., Zhang X., Hu H., Zhang J. (2021). Effects of cadmium stress on growth and physiological characteristics of sassafras seedlings. Sci. Rep..

[8] Rehman S., Abbas G., Shahid M., Saqib M., Farooq A.B.U., Hussain M., Farooq A. (2019). Effect of salinity on cadmium tolerance, ionic homeostasis and oxidative stress responses in conocarpus exposed to cadmium stresss. Ecotoxico. Environ..

[9] Mitsopoulou N., Lakiotis K., Golia E.E., Khah E.M., Pavli O.I. (2021). Response of hrpZPsph-transgenic N. benthamiana plants under cadmium stress. Environ. Sci. Pollut. Res..

[10] Kaya C. (2021). Salicylic acid-induced hydrogen sulphide improves lead stress tolerance in pepper plants by upraising the ascorbate-glutathione cycle. Physiol. Plantr..

[11] Guedes F.R.C.M., Maia C.F., da Silva B.R.S., Batista B.L., Alyemeni M.N., Ahmad P., da Silva Lobato A.K. (2021). Exogenous 24-Epibrassinolide stimulates root protection, and leaf antioxidant enzymes in lead stressed rice plants: Central roles to minimize Pb content and oxidative stress. Environ. Pollut..

[12] Abdal N., Abbas G., Asad S.A., Ghfar A.A., Shah G.M., Rizwan M., Ali S., Shahbaz M. (2021). Salinity mitigates cadmium-induced phytotoxicity in quinoa (*Chenopodium quinoa* Willd.) by limiting the Cd uptake and improved responses to oxidative stress: Implications for phytoremediation. Environ. Geochem. Health.

[13] Iftikhar A., Abbas G., Saqib M., Shabbir A., Amjad M., Shahid M., Ahmad I., Iqbal S., Qaisrani S.A. (2022). Salinity modulates lead (Pb) tolerance and phytoremediation potential of quinoa: A multivariate comparison of physiological and biochemical attributes. Environ. Geochem. Health.

[14] 14 Abbas G., Amjad M., Saqib M., Murtaza B., Asif Naeem M., Shabbir A., Murtaza G. (2021). Soil sodicity is more detrimental than salinity for quinoa (*Chenopodium quinoa* Willd.): A multivariate comparison of physiological, biochemical and nutritional quality attributes. J. Agron. Crop. Sci..

[15] Fatemi H., Pour B.E., Rizwan M. (2021). Foliar application of silicon nanoparticles affected the growth, vitamin C, flavonoid, and antioxidant enzyme activities of coriander (*Coriandrum sativum* L.) plants grown in lead (Pb)-spiked soil. Environ. Sci. Pollut. Res..

[16] Murtaza B., Naeem F., Shahid M., Abbas G., Shah N.S., Amjad M., Bakhat H.F., Imran M., Niazi N.K., Murtaza G. (2019). A multivariate analysis of physiological and antioxidant responses and health hazards of wheat under cadmium and lead stress. Environ. Sci. Pollut. Res..

[17] Amjad M., Iqbal M.M., Abbas G., Farooq A.B.U., Naeem M.A., Imran M., Murtaza B., Nadeem M., Jacobsen S.-E. (2021). Assessment of cadmium and lead tolerance potential of quinoa (Chenopodium quinoa Willd) and its implications for phytoremediation and human health. Environ. Geochem. Health.

[18] Parvez S., Abbas G., Shahid M., Amjad M., Hussain M., Asad S.A., Imran M., Naeem M.A. (2020). Effect of salinity on physiological, biochemical and photostabilizing attributes of two genotypes of quinoa (*Chenopodium quinoa* Willd.) exposed to arsenic stress. Ecotoxicol. Environ..

[19] Adrees M., Ali S., Rizwan M., Zia-ur-Rehman M., Ibrahim M., Abbas F., Farid M., Qayyum M.F., Irshad M.K. (2015). Mechanisms of silicon-mediated alleviation of heavy metal toxicity in plants: A review. Ecotoxicol. Environ. Saf..

[20] Ali S., Bharwana S.A., Rizwan M., Farid M., Kanwal S., Ali Q., Ibrahim M., Gill R.A., Khan M.D. (2015). Fulvic acid mediates chromium (Cr) tolerance in wheat (*Triticum aestivum* L.) through lowering of Cr uptake and improved antioxidant defense system. Environ. Sci. Pollut. Res..

[21] Cakmak I. (2005). The role of potassium in alleviating detrimental effects of abiotic stresses in plants. J. Plant. Nutr. Soil Sci..

[22] Abbas G., Rehman S., Siddiqui M.H., Ali H.M., Farooq M.A., Chen Y. (2022). Potassium and humic acid synergistically increase salt tolerance and nutrient uptake in contrasting wheat genotypes through ionic homeostasis and activation of antioxidant enzymes. Plants.

[23] Siddique M.H., Al-Whaibi A.H., Sakran M.O., Basalah H.M.A. (2012). Effect of calcium and potassium on antioxidant system of Vicia faba L. under cadmium stress. Int. J. Mol. Sci..

[24] Bybordi A. (2015). Influence of exogenous application of silicon and potassium on physiological responses, yield and yield components of salt-stressed wheat. Commun. Soil Sci. Plant Anal..

[25] Zorb C., Senbayramb M., Peiter E. (2014). Potassium in agriculture—Status and perspectives. J. Plant Physiol..

[26] Zaheer M.M., Yasin N.A., Ahmad S.R., Khan W.U., Ahmad A., Ali A., Rehman S.U. (2018). Amelioration of cadmium stress in gladiolus (*Gladiolus grandiflora* L.) by application of potassium and silicon. J. Plant Nutr..

[27] Alzahrani Y. (2018). The defensive role of silicon in wheat against stress conditions induced by drought, salinity or cadmium. Ecotoxicol. Environ. Saf..

[28] Ali M., Afzal S., Parveen A., Kamran M., Javed M.R., Abbasi G.H., Malik Z., Riaz M., Ahmad S., Chattha M.S. (2021). Silicon mediated improvement in the growth and ion homeostasis by decreasing Na^+^ uptake in maize (Zea mays L.) cultivars exposed to salinity stress. Plant Physiol. Biochem..

[29] Huang H., Rizwan M., Li M., Song F., Zhou S., He X., Ding R., Dai Z., Yuan Y., Cao M. (2019). Comparative efficacy of organic and inorganic silicon fertilizers on antioxidant response, Cd/Pb accumulation and health risk assessment in wheat (*Triticum aestivum* L.). Environ. Pollut..

[30] Khan A., Bilal S., Khan A.L., Imran M., Al-Harrasi A., Al-Rawahi A., Lee I.J. (2020). Silicon-mediated alleviation of combined salinity and cadmium stress in date palm (*Phoenix dactylifera* L.) by regulating physio-hormonal alteration. Ecotoxicol. Environ. Saf..

[31] Hoagland D.R., Arnon D.I. (1950). The water-culture method for growing plants without soil. Circular. Calif. Agric. Exp. Stn..

[32] Lichtenthaler H.K. (1987). Chlorophylls and carotenoids: Pigments of photosynthetic biomembranes. Methods Enzymol..

[33] Islam E., Liu D., Li T., Yang X., Jin X., Mahmood Q., Tian S., Li J. (2008). Effect of Pb toxicity on leaf growth, physiology and ultrastructure in the two ecotypes of *Elsholtzia argyi*. J. Hazard. Mater..

[34] Hodges D.M., DeLong J.M., Forney C.F., Prange R.K. (1999). Improving the thiobarbituric acid-reactive-substances assay for estimating lipid peroxidation in plant tissues containing anthocyanin and other interfering compounds. Planta.

[35] Sairam R.K., Rao K.V., Srivastava G.C. (2002). Differential response of wheat genotypes to long term salinity stress in relation to oxidative stress, antioxidant activity and osmolyte concentration. Plant Sci..

[36] Dhindsa R.S., Plumb-Dhindsa P., Thorpe T.A. (1981). Leaf senescence: Correlated with increased levels of membrane permeability and lipid peroxidation, and decreased levels of superoxide dismutase and catalase. J. Exp. Bot..

[37] Nakano Y., Asada K. (1981). Hydrogen peroxide is scavenged by ascorbate-specific peroxidase in spinach chloroplasts. Plant Cell Physiol..

[38] Aebi H. (1984). Catalase in vitro. Methods Enzymol..

[39] Hemeda H.M., Klein B.P. (1990). Effects of naturally occurring antioxidants on peroxidase activity of vegetable extracts. J. Food Sci..

[40] Steel R., Torrie J., Dickey D. (1997). Principles and Procedures of Statistics: A Biometrical Approach.

[41] Shabir R., Abbas G., Saqib M., Shahid M., Shah G.M., Akram M., Niazi N.K., Naeem M.A., Hussain M., Ashraf F. (2018). Cadmium tolerance and phytoremediation potential of acacia (*Acacia nilotica* L.) under salinity stress. Int. J. Phytorem..

[42] Gu H., Qiu H., Tian T., Zhan S., Deng T., Chaney R.L., Wang S., Tang Y., Morel J., Qiu R. (2011). Mitigation effects of silicon rich amendments on heavy metal accumulation in rice (*Oryza sativa* L.) planted on multi-metal contaminated acidic soil. Chemosphere.

[43] Chen R., Zhang C., Zhao Y., Huang Y., Liu Z., Chen R. (2018). Foliar application with nano-silicon reduced cadmium accumulation in grains by inhibiting cadmium translocation in rice plants. Environ. Sci. Pollut. Res..

[44] Huang F., Wen X., Cai Y., Cai K. (2018). Silicon-Mediated enhancement of heavy metal tolerance in rice at different growth stages. Int. J. Environ. Res. Public Health.

[45] Ke Y.C., Cao M., Yang X.F., Huang Z., Song L.Q., Liu Y. (2015). Effects of spraying different concentrations of organic silicon fertilizer on yield and quality of tropical melon. J. South. Agric..

[46] Ma J., Cai H., He C., Zhang W., Wang L. (2015). A hemicellulose-bound form of silicon inhibits cadmium ion uptake in rice (*Oryza sativa*) cells. New Phytol..

[47] Li X., Long J., Peng P., Chen Q., Dong X., Jiang K. (2018). Evaluation of calcium oxide of quicklime and SieCaeMg fertilizer for remediation of Cd uptake in rice plants and Cd mobilization in two typical Cd-polluted paddy soils. Int. J. Environ. Res..

[48] Zhang S., Ni X., Arif M., Zheng J., Stubbs A., Li C. (2020). NaCl improved Cd tolerance of the euhalophyte Suaeda glauca but not the recretohalophyte *Limonium aureum*. Plant Soil..

[49] Gallego S.M., Pena L.B., Barcia R.A., Azpilicueta C.E., Iannone M.F., Rosales E.P., Zawoznik M.S., Groppa M.D., Benavides M.P. (2012). Unravelling cadmium toxicity and tolerance in plants: Insight into regulatory mechanisms. Environ. Exp. Bot..

[50] Lu Y.G., Ma J., Teng Y., He J.Y., Christie P., Zhu L.J., Ren W.J., Zhang M.Y., Deng S.P. (2018). Effect of silicon on growth, physiology, and cadmium translocation of tobacco (*Nicotiana tabacum* L.) in cadmium-contaminated soil. Pedosph. Int. J..

[51] Song Z.Z., Duan C.L., Guo S.L., Yang Y., Feng Y.F., Ma R.J., Yu M.L. (2015). Potassium contributes to zinc stress tolerance in peach (Prunus persica) seedlings by enhancing photosynthesis and the antioxidant defense system. Genet. Mol. Res..

[52] Shahid M., Farooq A.B.U., Rabbani F., Khalid S., Dumat C. (2019). Risk assessment and biophysiochemical responses of spinach to foliar application of lead oxide nanoparticles: A multivariate analysis. Chemosphere.

[53] Munns R., Tester M. (2008). Mechanisms of salinity tolerance. Annu. Rev. Plant. Biol..

[54] Pourrut B., Shahid M., Dumat C., Winterton P., Pinelli E. (2011). Lead uptake, toxicity, and detoxification in plants. Rev. Environ. Contam. Toxicol..

[55] Li X., Zhang X., Wang X., Yang X., Cui Z. (2019). Bioaugmentation-assisted phytoremediation of lead and salinity co-contaminated soil by Suaeda salsa and *Trichoderma asperellum*. Chemosphere.

[56] Choi Y.E., Harada E., Wada M., Tsuboi H., Mortia Y., Kusano T., Sano H. (2001). Detoxification of cadmium in tobacco plant: Formation and active excretion of crystals containing cadmium and calcium through trichomes. Planta.

[57] Song W.Y., Martinoia E., Lee J., Kim D., Kim D.-Y., Vogt E., Shim D., Choi K.S., Hwang I., Lee Y. (2004). A novel family of cys-rich membrane proteins mediates cadmium resistance in Arabidopsis. Plant Physiol..

[58] Howladar S.M., Al-robai S.A., Al-zahrani F.S., Howladar M.M., Aldhebiani A.Y. (2018). Silicon and its application method effects on modulation of cadmium stress responses in *Triticum aestivum* (L.) through improving the antioxidative defense system and polyamine gene expression. Ecotoxicol. Environ. Saf..

[59] Kim Y.H., Khan A.L., Waqas M., Lee I.J. (2017). Silicon regulates antioxidant activities of crop plants under abiotic-induced oxidative stress: A review. Front. Plant Sci..

[60] Emamverdian A., Ding Y., Xie Y., Sangari S. (2018). Silicon mechanisms to ameliorate heavy metal stress in plants. BioMed Res. Int..

[61] Imtiaz M. (2016). Silicon occurrence, uptake, transport and mechanisms of heavy metals, minerals and salinity en hanced tolerance in plants with future prospects: A review. J. Environ. Manag..

[62] Souri Z., Karimi N., de Oliveira L.M. (2018). Antioxidant enzymes responses in shoots of arsenic hyperaccumulator, *Isatis cappadocica* Desv., under interaction of arsenate and phosphate. Environ. Technol..

[63] Abbaspour A., Kalbasi M., Hajrasuliha S., Fotovat A. (2008). Effect of organic matter and salinity on ethylenediaminetetraacetic acid–extractable and solution species of cadmium and lead in three agricultural soils. J. Commun. Soil. Sci. Plant Anal..

